# Insights on the Proteases Involved in Barley and Wheat Grain Germination

**DOI:** 10.3390/ijms20092087

**Published:** 2019-04-28

**Authors:** Mercedes Diaz-Mendoza, Isabel Diaz, Manuel Martinez

**Affiliations:** 1 Centro de Biotecnologia y Genomica de Plantas (CBGP, UPM-INIA), Universidad Politecnica de Madrid (UPM)- Instituto Nacional de Investigacion y Tecnología Agraria y Alimentaria (INIA), Campus Montegancedo, 28223 Pozuelo de Alarcon, Madrid, Spain; mercedes.diaz.mendoza@upm.es (M.D.-M.); i.diaz@upm.es (I.D.); 2 Departamento de Biotecnologia-Biologia Vegetal, Escuela Tecnica Superior de Ingenieria Agronomica, Alimentaria y de Biosistemas, UPM, 28040-Madrid, Spain

**Keywords:** barley, wheat, protease, germination, grain

## Abstract

Seed storage proteins must be hydrolyzed by proteases to deliver the amino acids essential for embryo growth and development. Several groups of proteases involved in this process have been identified in both the monocot and the dicot species. This review focuses on the implication of proteases during germination in two cereal species, barley and wheat, where proteolytic control during the germination process has considerable economic importance. Formerly, the participation of proteases during grain germination was inferred from reports of proteolytic activities, the expression of individual genes, or the presence of individual proteins and showed a prominent role for papain-like and legumain-like cysteine proteases and for serine carboxypeptidases. Nowadays, the development of new technologies and the release of the genomic sequences of wheat and barley have permitted the application of genome-scale approaches, such as those used in functional genomics and proteomics. Using these approaches, the repertoire of proteases known to be involved in germination has increased and includes members of distinct protease families. The development of novel techniques based on shotgun proteomics, activity-based protein profiling, and comparative and structural genomics will help to achieve a general view of the proteolytic process during germination.

## 1. Introduction

Barley is considered a model organism for the investigation of the cereal germination process. Along with maize and rice, allohexaploid bread wheat and diploid barley are the most cultivated crops in the world (FAOSTAT database, http://www.fao.org/faostat, access on 22 April 2019). Their economic importance and close relationship support a parallel study of both cereals. The role of plant proteases in the mobilization of storage proteins that have accumulated in seeds has been largely established in both the dicotyledonous and the monocotyledonous species [1,2,3]. Storage proteins must be degraded to sustain embryo growth and development until an autotrophic growth is reached. Thus, a controlled proteolysis is crucial for the accurate delivery of amino acids in the initial stages of seed germination. Several protease families are involved in the germination process. Cysteine proteases (CysProt) of the C1A family, which are known as papain-like, and the C13 family, alternatively called legumains or vacuolar processing enzymes (VPEs), are the main proteases involved in the germination of both dicot and monocot species [1,2,4]. In dicot species, storage proteins are placed in the mesophyll of the cotyledons and in the embryonic axis. Members of the papain-like, legumain-like, and subtilisin-like (S8) families have been demonstrated to participate in the breakdown and mobilization of reserve proteins from seeds to cotyledons during germination [5,6,7].

Monocot seeds include proteins with many different functions. Around 80% of these proteins are storage proteins, packed in the endosperm together with starch and lipids. These proteins are synthesized during grain development and maturation and consequently are involved in germination. Among the proteases involved in the germination process, CysProt are responsible for around 90% of the proteolytic activity [8]. Other than CysProt from the papain family (C1A) and the legumain family (C13), members of the S10 serine carboxypeptidases (SCP) have also been implicated in the germination process in cereal grains. Papain-like CysProt participating in different stages of the germination process include the cathepsin L-like proteases identified in rice (oryzains α and β) and triticale (EP8), the cathepsin H-like proteases (oryzain y) from rice, and the cathepsin B-like proteases (BdCathB) from *Brachypodium distachyon* [9,10,11,12]. Among legumains, the OsVPE-1 protease was described in the degradation of stored proteins in the rice grain [13], and the REP-2 rice legumain was suggested as an activator of other CysProt during rice germination [14]. In this process, the SCP46 serine carboxypeptidase from rice regulates grain filling and seed germination upon hormonal induction [15,16]. Besides, serine carboxypeptidases I and III from triticale grains effectively degraded storage proteins that were proteolytically modified by the cathepsin L-like protease EP8 [17,18].

The participation of proteases in the germination processes of barley and wheat will be widely described in following sections.

## 2. Mobilization of Stored Proteins During the Germination of Barley and Wheat

**Figure 1:**
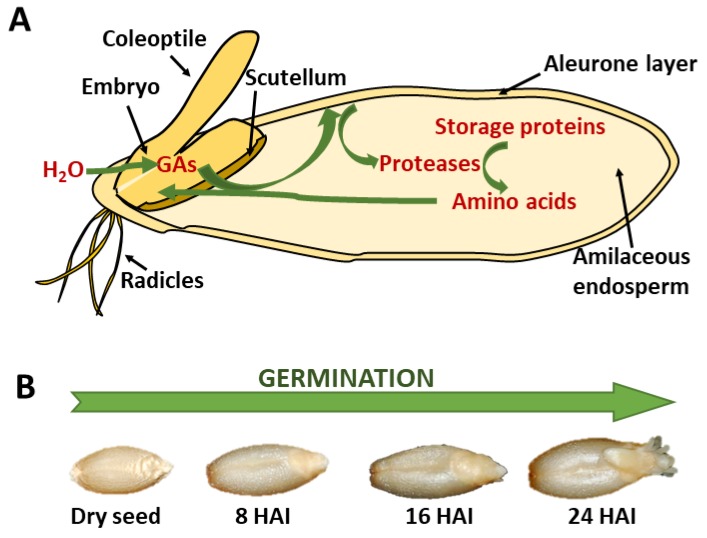
Cereal seed germination. (**A**) Schematic representation of the main events during storage protein remobilization in cereal seeds. (**B**) Photographs of the germination process of barley grains. HAI, hours after imbibition.

Monocot species like barley and wheat have caryopses or cereal grains as propagation units. Cereal grains are endospermic seeds, meaning that the storage proteins are accumulated in the endosperm tissue (Figure 1A). This tissue consists of the starchy endosperm, which is a dead storage tissue, and the aleurone layer, which is formed by living cells. The main tissues found in the barley embryo are the coleoptile, the scutellum, and the radicle. During seed development and maturation, deposition of reserves within the storage tissue takes place. Starch, proteins, and lipids are mainly accumulated in the endosperm tissue but are also found in axis organs like the radicle and the embryonic shoot, or in the outer aleurone layer [19]. Cereal grains contain relatively little protein, with an average stored amount of 10–12% of the dry weight [19]. This storage fraction represents 80–85% of the total protein content. The main seed storage proteins are classified as albumins, globulins, and prolamins, on the basis of their solubility [20]. Most storage proteins in barley and wheat are prolamins [19], which are named hordeins in barley and gliadins in wheat. The antagonism between gibberellins (GA) and abscisic acid (ABA) is an important factor regulating the developmental transition from seed maturation to seed germination [21]. In terms of physiological and morphological changes, seed germination typically begins with dry mature seed imbibition and ends with radicle protrusion (Figure 1B). The embryo is responsible for the synthesis of GA after imbibition in water. This hormone reaches the aleurone layer via the scutellum, where it induces the expression of genes encoding α-amylases and proteases. The combination of the CysProt stored in the protein bodies and de novo-formed proteolytic enzymes, which are spread into the endosperm, triggers the hydrolysis of most proteins in the storage tissue. The resulting amino acids and small peptides are absorbed by the scutellum, which delivers them to the growing seedling (Figure 1A). Once the radicle breaks and protrudes from the seed coat, the germination process is accomplished [22].

## 3. Investigation of Proteases in the Germination of Barley and Wheat before High-Throughput Technologies

**Figure 2:**
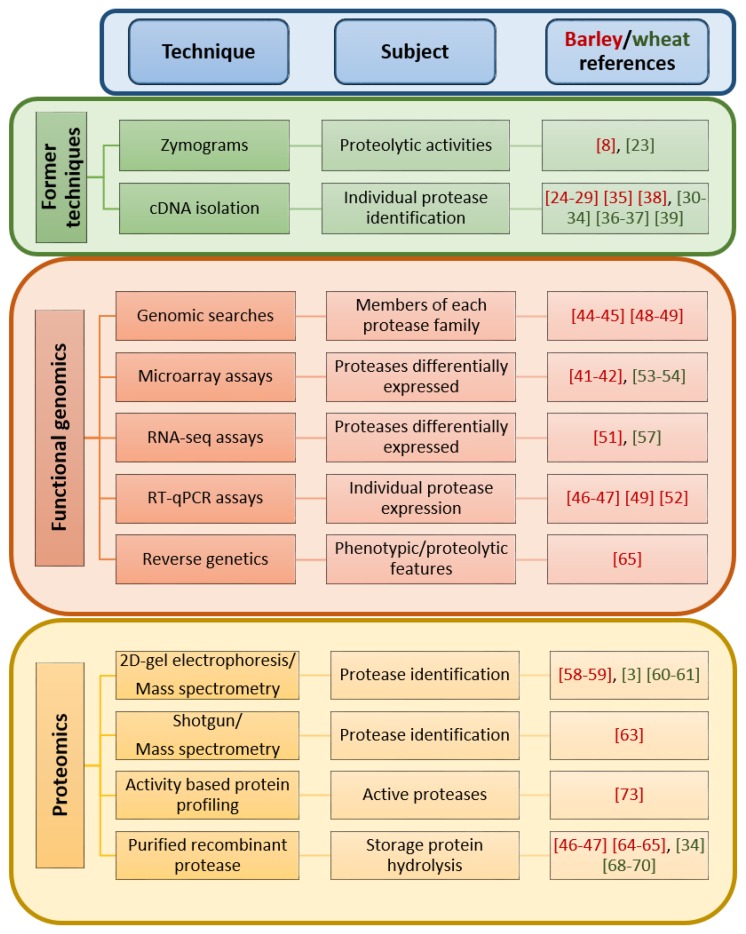
Global scheme of the main techniques used to analyze the implication of proteases during the germination of barley and wheat grains.

Historically, the participation of proteases during grain germination was inferred from reports on proteolytic activities, the expression of individual genes during the process, or the presence of individual proteins in the germinating grain (Figure 2). Zhang and Jones [8], using bidimensional gel analyses and class-specific protease inhibitors, found 42 different spots with protease activity in the barley germinating grain, putatively corresponding to 27 cysteine proteases, 8 serine proteases, 4 aspartic proteases, and 3 metalloproteases. Similarly, Dominguez and Cejudo [23] used polyacrylamide gels copolymerized with gelatin to detect proteolytic activities in germinating wheat grains. Again, putative CysProt activity was preferentially observed, although serine, aspartic, and metalloprotease activities were also present. In parallel, individual proteases were isolated from barley and wheat germinating grains and identified by sequencing the corresponding cDNAs. The first barley CysProt described participating in the proteolytic degradation of the storage proteins in the grains [24,25,26,27] were the cathepsin L-like proteases EP-A and EP-B from the C1A family. Besides, a cathepsin H-like protease, aleurain, and a cathepsin B-like protein, HvCathB, were detected in the aleurone of the barley grain [28,29]. In wheat, several cathepsin L-like CysProt, WEB-1, WEP-2, EP-A, WCP-2, and gliadain, and the cathepsin B-like proteases Al16, Al20, and Al21 were described as participants in the proteolysis processes of the germinating grain [30,31,32,33,34]. In addition, members of the S10 serine carboxypeptidase family were implicated in the germination of barley and wheat [35,36,37], and some aspartic proteases were identified in barley and wheat seeds [38,39].

Nowadays, advances in high-throughput sequencing have resulted in the development of new technologies and strategies that give support to experimental research. The release of the genomic sequences of wheat and barley has permitted the application of genome-scale approaches, such as those used in functional genomics and proteomics. These technologies have been employed, mainly in barley, in the study of proteases during the germination process (Figure 2).

## 4. Functional Genomic-Based Advances in the Identification of Proteases in the Germination of Barley and Wheat Grains

The extremely large number of expressed sequence tags (EST) and cDNA sequences in barley permitted the first approximation of the protease repertoire. Using the Affymetrix Barley1 GeneChip 22K [40], the expression of about 20 C1A proteases could be checked. This GeneChip has been used in several studies on gene expression during germination and seedling elongation (reviewed in [41]). Among these studies, a detailed transcriptome analysis of barley grain germination can be highlighted [42]. In this analysis, several C1A papain-like CysProt, such as *EP-A*, *EP-B1*, and *EP-B2*, were expressed in both the aleurone and the embryo during grain germination. In addition, transcripts of cathepsin B-like (*HvPap-19* and *-20*), cathepsin H-like (*aleurain*), cathepsin F-like (*HvPap-1*), and some cathepsin L-like proteases (*HvPap-4* and *-6*) were abundant during seed germination in both the aleurone and the embryo, but were also expressed during seed maturation. Besides, transcripts for several serine carboxypeptidases were highly detected during germination in both the embryo and the aleurone [42].

A second step in the identification process came from the public release of the barley genome sequence [43]. Previous analysis from EST collections showed the presence of 32 C1A papain-like members [44], which increased to 41 members upon genomic mining [45]. A former analysis of the expression of cathepsin-L like proteases from different phylogenetic groups showed that four of the five selected cathepsin L-like genes (*HvPap-4*, *HvPap-6*, *HvPap-10*, and *HvPap-17*) were primarily transcribed in germinating embryos [46]. The most abundant transcripts were those of *HvPap-10*, which had a seed-specific pattern of expression, followed by *HvPap-6* and *HvPap-4*. Whereas a lower expression of the *HvPap-17* gene was detected in germinating embryos, *HvPap-16* was exclusively transcribed in barley leaves. No expression of any of the cathepsin L-like genes studied was detected in the developing barley endosperms. During germination, GA treatment of the aleurone layer increased the quantity of transcripts from *HvPap-6* and *HvPap-10* but had no effect on the expression level of the *HvPap-4* gene. Likewise, de-embryonated barley grains showed that the *HvPap-1* gene was expressed during grain germination and that GA treatment induced a remarkable increase in its expression [47].

The genomic content of C13 legumain-like genes has also been addressed. Formerly, five legumains were reported from EST collections [44], two additional legumains were later detected [48], and finally, a novel protein was added after an in-depth search of the barley genome [49]. Regarding the barley legumains group, it has been suggested that legumains are able to process other CysProt in order to activate them to take part in the proteolytic degradation of the storage proteins [50]. For example, the HvLeg-2 legumain of barley, which is highly expressed during germination, could be involved in the mobilization of storage proteins, either by direct proteolytic degradation or by the processing and activation of other CysProt [49,50]. In fact, the capacity of HvLeg-2 to degrade storage seed globulins was demonstrated, confirming its role as a hydrolytic enzyme against storage proteins. Likewise, HvLeg-2 could participate in the processing of other peptidases, such as the papain-like CysProt induced by GA, which degrade hordeins [46,47]. This is similar to the action of the legumain REP-2 on the papain-like peptidase REP-1 in germinating rice seeds [14].

Recently, a new methodology has been developed for isolating fragments of aleurone, starchy endosperm, embryo, scutellum, pericarp–testa, husk, and crushed cell layers from barley germinated grain [51]. This method is based on rapid fixation of the intact grain, followed by dissection for subsequent transcriptomic analyses. Using this technology, the expression profiles of many genes were precisely defined during the first 24 h of germination [51]. Interestingly, an analysis of the differential expressed genes (DEG) in the aleurone fragment nearest the embryo after 24 h of germination showed the induction of many different proteases, including papain-like CysProt, legumain-like CysProt, and serine carboxypeptidases, and also several aspartic proteases, metalloproteases, and subtilisin-like serine proteases. Many of these proteases were exclusively upregulated in this proximal part of the aleurone layer, and none of them were overexpressed in the distal fragment of the aleurone. Following 24 h of germination, the set of upregulated proteases in the embryo and scutellum comprised several proteases exclusively upregulated in these tissues and some of the proteases overexpressed in the aleurone. Recently, the upregulation of several subtilisin-like serine proteases during grain germination has been confirmed by RT-qPCR assays [52].

In contrast to wide knowledge on the gene expression in the barley germinating grain, studies on wheat are scarce. To date, transcriptome expression profiles during seed germination have been performed using the GeneChip^®^ Wheat Genome Array [53,54]. In whole germinating grains, the expression of several cysteine, aspartic, and serine proteases increased significantly between 24 h and 48 h after imbibition, with a peak at 36 h for the most expressed class, the CysProt [54]. Efforts on optimizing RNA-seq analyses clashed with the poor quality of the first draft of the wheat genome released by the International Wheat Genome Sequencing Consortium [55] (IWGSC, 2014). However, a tremendous effort using novel approaches generated the recently published high-quality linear reference assembly, IWGSC RefSeq v1.0 [56]. Likewise, many RNA-seq analyses have been performed and implemented in different portals, such as the Wheat Expression Browser (www.wheat-expression.com, [57]) which includes 850 wheat RNA-sequencing samples derived from 32 tissues at different growth stages and/or challenged by different stress treatments. Unfortunately, these analyses did not cover the germinating grain.

## 5. Proteomic-Based Advances on Proteases in the Germination of Barley and Wheat Grains

Wide proteomic analyses of barley and wheat grains were formerly based on two-dimensional gel electrophoresis, which was subsequently coupled with mass spectrometry. In the absence of a genome sequence, barley gene and EST sequences, combined with information from other cereals, facilitated the identification of barley proteins. From this wide range of proteomic analyses, many barley seed proteins were identified (reviewed by [58]). Besides, to avoid the masking of low-abundant proteins by large amounts of starchy endosperm storage proteins, isolated aleurone layers were used in proteomic analyses. When treated with GA, several C1A cysteine proteases, S10 serine proteases, and A1 aspartic proteases were identified in this tissue [59].

In contrast, relatively few proteomic studies have been performed during wheat seed germination. Several analyses combined two-dimensional electrophoresis (2-DE) with MALDI –TOF/TOF MS to explore the proteomic changes in the embryo and endosperm that occur throughout the germination period. From these analyses, distinct differentially expressed proteins were found in the seed embryo and endosperm, which presumably cooperate in seed germination [3,60,61]. Although the authors claimed that some of these proteins were related to storage protein metabolism, no individual proteases were identified. Besides, the proteome of isolated aleurone layers has only been addressed during seed development [62]. Recently, a gel-free proteomics approach was performed to obtain a dynamic proteome survey during barley malting. This shotgun proteomic technique entails the in-solution tryptic digestion of precipitated proteins and an analysis of peptides by nanoLC–MS/MS. A high number of proteins were identified [63], including several aspartic, cysteine, metallo, and serine peptidases, which demonstrates the strength of this technique for identifying low-expressed proteins. Although similar proteomics approaches have been performed in wheat, no one has analyzed grain germination.

On the other hand, protease activity in the barley grain has been analyzed using two different approaches. A classical approach is based on the capacity of recombinant proteases to degrade storage compounds. In a first analysis, whereas recombinant HvPap-10 protease was able to completely degrade all electrophoretic bands corresponding to B, C, and D hordeins from grain extracts, HvPap-6 only partially reduced the presence of those bands [46]. In addition, the capacity of HvPap-10 to hydrolyze the recombinant hordeins (B1, B3, and γ1) expressed in *Escherichia coli* was tested. After 2 h of incubation with HvPap-10, an almost complete degradation of γ1 and a partial digestion of hordein B1 and B3 were observed [64]. Besides, recombinant HvPap-1 was able to degrade different barley proteins (hordeins, albumins, and globulins) stored in the barley endosperm [47]. Further insight on the role of proteases in grain germination in transgenic plants was provided by silencing or overexpressing a specific protease. Only the silencing or overexpressing of the cathepsin F-like *HvPap-1* protease by barley plants has been described. These plants showed differential accumulation of storage molecules such as starch, proteins, and free amino acids in the grain, as well as disturbed electrophoretic patterns of hordeins, globulins, and albumins during the germinating process. Silencing lines showed a drastic delay in the grain germination process. Remarkably, this phenotypic feature could not be directly related to cathepsin F-like deficiencies, as alterations in the cathepsin L/F-like proteolytic activities were also accompanied by changes in cathepsin B-like and trypsin-like proteolytic activities [65].

In wheat, the C1A protease gliadain, purified from *E. coli*, was able to hydrolyze the storage α, β, and γ gliadins, but not the glutenins from grain extracts [34]. Gluten is a heterogeneous mixture of insoluble storage proteins, called gliadins, which contain proline-rich and glutamine-rich repetitive sequences. The fact that several of the peptides derived from gluten are toxic for humans with celiac disease led to the identification of proteases with the ability to degrade it [66,67]. Proteases from protein extracts of germinated barley and wheat grains showed the ability to degrade gliadin-derived toxic peptides [68]. In particular, the C1A protease Triticain-α, formerly thought to participate in seed germination by digesting storage proteins [69], was shown to possess glutenase activity in vitro [70]. Triticain-α cleavage sites were found in the majority of the previously identified gluten-derived toxic peptides, including the major 33-mer α-gliadin-derived peptide. These findings support the potential of Triticain-α as a basic compound for the development of drugs against celiac disease [70].

The second approach is based on the development of activity-based proteomics, also known as activity-based protein profiling (ABPP). This method uses molecular probes which bind irreversibly to the reactive site of members of specific groups of enzymes. The results provided information on enzyme activity, not just protein abundance [71], allowing differentiation between the inactive plant proteases synthesized as zymogens and the active proteases, after proteolytic processing. As the specificity of many commercial protease inhibitors is inaccurate, specific fluorescent probes were developed for ABPP. When applied on *Arabidopsis* germinating seeds, the fluorescent activity-based probes specifically targeted three distinct cysteine protease subfamilies, revealing the dynamic activities of aleurain-like proteases, cathepsin B-like proteases, and vacuolar processing enzymes during the remobilization of stored proteins [72]. This technology has recently been applied to monitor the activity of different enzymes in the germination process of the barley grain. Using specific probes and ABPP to detect the active enzymes extracted from the aleurone layers of a commercial malting barley variety, several active proteases were found to be induced by GA, such as putative aleurains, cathepsin- B-like proteases, and serine hydrolases [73].

## 6. Conclusions and Future Perspectives

Germination is a key process in the life cycle of plants. During this period, a new plant starts its development from the embryo with the help of the compounds stored in the seed endosperm. The continuous release of nutrients from the endosperm to the embryo is crucial to achieve the correct development of the new plant and to avoid agronomical losses due to the absence of seed germination in the field. Therefore, knowledge on the regulatory mechanisms that take part during this process must be improved to establish the best conditions for a correct germination. In the case of barley and wheat, many advances have been made in understanding the role of proteases in the grain due to their potential value for the brewing industry and their relationship with the celiac disease. Using different technologies, many proteases have been identified and closely associated to the grain germination. The development of novel techniques based on shotgun proteomics, ABPP, and comparative and structural genomics will lead to a more comprehensive understanding of this process and the specific roles of the proteases involved. For example, the identification by ABPP of active proteases during the barley grain germination process will increase the selection of efficient prolamin-degrading proteases. To reduce costs, the brewing industry is replacing barley malt with unmalted grains [74]. Thus, these proteases may be included in the commercial brewing enzyme cocktails used to improve the wort obtained from unmalted barley grains. Likewise, technical advances will allow the selection of high gliadin-degrading efficient proteases, which could be combined with the low-gliadin wheat plants obtained by genome edition [75], as therapeutic alternatives in the treatment of celiac disease. Furthermore, silencing or overexpressing a specific protease will contribute to the knowledge on the role of the protease and also in understanding the intricate network of proteolytic reactions combining different classes of proteases and protease inhibitors involved in the remobilization of storage proteins. The required technology has recently been developed, and the challenge is to combine research efforts to address key questions concerning the control of proteolysis during seed germination.

## References

[1] Grudkowska M., Zagdańska B. (2004). Multifunctional role of plant cysteine proteinases. Acta Biochim. Pol..

[2] Szewińska J., Simińska J., Bielawski W. (2016). The roles of cysteine proteases and phytocystatins in development and germination of cereal seeds. J. Plant Physiol..

[3] Liu Y., Han C., Deng X., Liu D., Liu N., Yan Y. (2018). Integrated physiology and proteome analysis of embryo and endosperm highlights complex metabolic networks involved in seed germination in wheat (*Triticum aestivum* L.). J. Plant Physiol..

[4] Tan-Wilson A.L., Wilson K.A. (2012). Mobilization of seed protein reserves. Physiol. Plant.

[5] Fischer J., Becker C., Hillmer S., Horstmann C., Neubohn B., Schlereth A., Senyuk V., Shutov A., Müntz K. (2000). The families of papain- and legumain-like cysteine proteinases from embryonic axes and cotyledons of Vicia seeds: Developmental patterns, intracellular localization and functions in globulin proteolysis. Plant Mol. Biol..

[6] Schlereth A., Becker C., Horstmann C., Tiedemann J., Müntz K. (2000). Comparison of globulin mobilization and cysteine proteinases in embryonic axes and cotyledons during germination and seedling growth of vetch (*Vicia sativa* L.). J. Exp. Bot..

[7] Toyooka K., Okamoto T., Minamikawa T. (2000). Mass transport of proform of a KDEL-tailed cysteine proteinase (SH-EP) to protein storage vacuoles by endoplasmic reticulum-derived vesicle is involved in protein mobilization in germinating seeds. J. Cell Biol..

[8] Zhang N., Jones B.L. (1995). Characterization of germinated barley endoproteolytic enzymes by two dimensional gel electrophoresis. J. Cereal Sci..

[9] Watanabe H., Abe K., Emori Y., Hosoyama H., Arai S. (1991). Molecular cloning and gibberellin-induced expression of multiple cysteine proteinases of rice seeds (oryzains). J. Biol. Chem..

[10] Prabucka B., Bielawski W. (2004). Purification and partial characteristic of a major gliadin-degrading cysteine endopeptidase from germinating triticale seeds. Acta Physiol. Plant..

[11] Prabucka B., Drzymała A., Grabowska A. (2013). Molecular cloning and expression analysis of the main gliadin-degrading cysteine endopeptidase EP8 from triticale. J. Cereal Sci..

[12] González-Calle V., Iglesias-Fernández R., Carbonero P., Barrero-Sicilia C. (2014). The BdGAMYB protein from Brachypodium distachyon interacts with BdDOF24 and regulates transcription of the BdCathB gene upon seed germination. Planta.

[13] Wang Y., Zhu S., Liu S., Jiang L., Chen L., Ren Y., Han X., Liu F., Ji S., Liu X. (2009). The vacuolar processing enzyme OsVPE1 is required for efficient glutelin processing in rice. Plant J..

[14] Kato H., Sutoh K., Minamikawa T. (2003). Identification, cDNA cloning and possible roles of seed-specific rice asparaginyl endopeptidase, REP-2. Planta.

[15] Washio K., Ishikawa K. (1994). Organ-specific and hormone-dependent expression of genes for serine carboxypeptidases during development and following germination of rice grains. Plant Physiol..

[16] Li Z., Tang L., Qiu J., Zhang W., Wang Y., Tong X., Wei X., Hou Y., Zhang J. (2016). Serine carboxypeptidase 46 Regulates Grain Filling and Seed Germination in Rice (*Oryza sativa* L.). PLoS ONE.

[17] Drzymała A., Bielawski W. (2009). Isolation and characterization of carboxypeptidase III from germinating triticale grains. Acta Biochim. Biophys. Sin..

[18] Drzymała A., Prabucka B., Bielawski W. (2012). Carboxypeptidase I from triticale grains and the hydrolysis of salt-soluble fractions of storage proteins. Plant Physiol. Biochem..

[19] Shewry P.R., Halford N.G. (2002). Cereal seed storage proteins: Structures, properties and role in grain utilization. J. Exp. Bot..

[20] Shewry P.R., Napier J.A., Tatham A.S. (1995). Seed storage proteins: Structures and biosynthesis. Plant Cell.

[21] Finch-Savage W.E., Leubner-Metzger G. (2006). Seed dormancy and the control of germination. New Phytol..

[22] Bewley J.D. (1997). Seed Germination and Dormancy. Plant Cell.

[23] Dominguez F., Cejudo F.J. (1996). Characterization of the Endoproteases Appearing during Wheat Grain Development. Plant Physiol..

[24] Poulle M., Jones B.L. (1988). A Proteinase from Germinating Barley: I. Purification and Some Physical Properties of a 30 kD Cysteine Endoproteinase from Green Malt. Plant Physiol..

[25] Koehler S.M., Ho T.H. (1990). A major gibberellic Acid-induced barley aleurone cysteine proteinase which digests hordein: Purification and characterization. Plant Physiol..

[26] Koehler S., Ho T.H. (1988). Purification and characterization of gibberellic Acid-induced cysteine endoproteases in barley aleurone layers. Plant Physiol.

[27] Mikkonen A., Porali I., Cercos M., Ho T.H. (1996). A major cysteine proteinase, EPB, in germinating barley seeds: Structure of two intronless genes and regulation of expression. Plant Mol. Biol..

[28] Rogers J.C., Dean D., Heck G.R. (1985). Aleurain: A barley thiol protease closely related to mammalian cathepsin H. Proc. Natl. Acad. Sci. USA.

[29] Martinez M., Rubio-Somoza I., Carbonero P., Diaz I. (2003). A cathepsin B-like cysteine protease gene from Hordeum vulgare (gene CatB) induced by GA in aleurone cells is under circadian control in leaves. J. Exp. Bot..

[30] Cejudo F.J., Murphy G., Chinoy C., Baulcombe D.C. (1992). A gibberellin-regulated gene from wheat with sequence homology to cathepsin B of mammalian cells. Plant J..

[31] Jivotovskaya A.V., Horstmann C., Vaintraub I.A. (1997). Detection of the isoenzymes of wheat grain proteinase A. Phytochemistry.

[32] Sutoh K., Kato H., Minamikawa T. (1999). Identification and possible roles of three types of endopeptidase from germinated wheat seeds. J. Biochem..

[33] Tsuji A., Tsuji M., Takami H., Nakamura S., Matsuda Y. (2004). Molecular cloning and expression analysis of novel wheat cysteine protease. Biochim. Biophys. Acta.

[34] Kiyosaki T., Matsumoto I., Asakura T., Funaki J., Kuroda M., Misaka T., Arai S., Abe K. (2007). Gliadain, a gibberellin-inducible cysteine proteinase occurring in germinating seeds of wheat, *Triticum aestivum* L., specifically digests gliadin and is regulated by intrinsic cystatins. FEBS J..

[35] Dal Degan F., Rocher A., Cameron-Mills V., von Wettstein D. (1994). The expression of serine carboxypeptidases during maturation and germination of the barley grain. Proc. Natl. Acad. Sci. USA.

[36] Dominguez F., Cejudo F.J. (1999). Patterns of starchy endosperm acidification and protease gene expression in wheat grains following germination. Plant Physiol..

[37] Domínguez F., González M.C., Cejudo F.J. (2002). A germination-related gene encoding a serine carboxypeptidase is expressed during the differentiation of the vascular tissue in wheat grains and seedlings. Planta.

[38] Törmäkangas K., Runeberg-Roos P., Ostman A., Tilgmann C., Sarkkinen P., Kervinen J., Mikola L., Kalkkinen N. (1991). Aspartic proteinase from barley seeds is related to animal cathepsin D. Adv. Exp. Med. Biol..

[39] Tamura T., Terauchi K., Kiyosaki T., Asakura T., Funaki J., Matsumoto I., Misaka T., Abe K. (2007). Differential expression of wheat aspartic proteinases, WAP1 and WAP2, in germinating and maturing seeds. J. Plant Physiol..

[40] Close T.J., Wanamaker S.I., Caldo R.A., Turner S.M., Ashlock D.A., Dickerson J.A., Wing R.A., Muehlbauer G.J., Kleinhofs A., Wise R.P. (2004). A new resource for cereal genomics: 22K barley GeneChip comes of age. Plant Physiol..

[41] Daneri-Castro S.N., Svensson B., Roberts T.H. (2016). Barley germination: Spatio-temporal considerations for designing and interpreting ‘omics’ experiments. J. Cereal Sci..

[42] Sreenivasulu N., Usadel B., Winter A., Radchuk V., Scholz U., Stein N., Weschke W., Strickert M., Close T.J., Stitt M. (2008). Barley grain maturation and germination: Metabolic pathway and regulatory network commonalities and differences highlighted by new MapMan/PageMan profiling tools. Plant Physiol..

[43] Mayer K.F., Waugh R., Brown J.W., Schulman A., Langridge P., Platzer M., Fincher G.B., Muehlbauer G.J., Sato K., Close T.J. (2012). A physical, genetic and functional sequence assembly of the barley genome. Nature.

[44] Martinez M., Diaz I. (2008). The origin and evolution of plant cystatins and their target cysteine proteinases indicate a complex functional relationship. BMC Evol. Biol..

[45] Diaz-Mendoza M., Velasco-Arroyo B., Gonzalez-Melendi P., Martinez M., Diaz I. (2014). C1A cysteine protease-cystatin interactions in leaf senescence. J. Exp. Bot..

[46] Martinez M., Cambra I., Carrillo L., Diaz-Mendoza M., Diaz I. (2009). Characterization of the Entire Cystatin Gene Family in Barley and Their Target Cathepsin L-Like Cysteine-Proteases, Partners in the Hordein Mobilization during Seed Germination. Plant Physiol..

[47] Cambra I., Martinez M., Dader B., Gonzalez-Melendi P., Gandullo J., Santamaria M.E., Diaz I. (2012). A cathepsin F-like peptidase involved in barley grain protein mobilization, HvPap-1, is modulated by its own propeptide and by cystatins. J. Exp. Bot..

[48] Radchuk V., Weier D., Radchuk R., Weschke W., Weber H. (2011). Development of maternal seed tissue in barley is mediated by regulated cell expansion and cell disintegration and coordinated with endosperm growth. J. Exp. Bot..

[49] Julian I., Gandullo J., Santos-Silva L.K., Diaz I., Martinez M. (2013). Phylogenetically distant barley legumains have a role in both seed and vegetative tissues. J. Exp. Bot..

[50] Cambra I., Garcia F.J., Martinez M. (2010). Clan CD of cysteine peptidases as an example of evolutionary divergences in related protein families across plant clades. Gene.

[51] Betts N.S., Berkowitz O., Liu R., Collins H.M., Skadhauge B., Dockter C., Burton R.A., Whelan J., Fincher G.B. (2017). Isolation of tissues and preservation of RNA from intact, germinated barley grain. Plant J..

[52] Galotta M.F., Pugliese P., Gutiérrez-Boem F.H., Veliz C.G., Criado M.V., Caputo C., Echeverria M., Roberts I.N. (2019). Subtilase activity and gene expression during germination and seedling growth in barley. Plant Physiol. Biochem..

[53] Yu Y., Zhen S., Wang S., Wang Y., Cao H., Zhang Y., Li J., Yan Y. (2016). Comparative transcriptome analysis of wheat embryo and endosperm responses to ABA and H2O2 stresses during seed germination. BMC Genom..

[54] Yu Y., Guo G., Lv D., Hu Y., Li J., Li X., Yan Y. (2014). Transcriptome analysis during seed germination of elite Chinese bread wheat cultivar Jimai 20. BMC Plant Biol..

[55] The International Wheat Genome Sequencing Consortium (IWGSC) (2014). A chromosome-based draft sequence of the hexaploid bread wheat (*Triticum aestivum*) genome. Science.

[56] Appels R., Eversole K., Feuillet C., Keller B., Rogers J., Stein N., Pozniak C.J., Choulet F., Distelfeld A., Poland J. (2018). Shifting the limits in wheat research and breeding using a fully annotated reference genome. Science.

[57] Ramírez-González R.H., Borrill P., Lang D., Harrington S.A., Brinton J., Venturini L., Davey M., Jacobs J., van Ex F., Pasha A. (2018). The transcriptional landscape of polyploid wheat. Science.

[58] Finnie C., Svensson B. (2009). Barley seed proteomics from spots to structures. J. Proteom..

[59] Finnie C., Andersen B., Shahpiri A., Svensson B. (2011). Proteomes of the barley aleurone layer: A model system for plant signalling and protein secretion. Proteomics.

[60] Dong K., Zhen S., Cheng Z., Cao H., Ge P., Yan Y. (2015). Proteomic Analysis Reveals Key Proteins and Phosphoproteins upon Seed Germination of Wheat (*Triticum aestivum* L.). Front. Plant Sci..

[61] He M., Zhu C., Dong K., Zhang T., Cheng Z., Li J., Yan Y. (2015). Comparative proteome analysis of embryo and endosperm reveals central differential expression proteins involved in wheat seed germination. BMC Plant Biol..

[62] Nadaud I., Tasleem-Tahir A., Chateigner-Boutin A.L., Chambon C., Viala D., Branlard G. (2015). Proteome evolution of wheat (*Triticum aestivum* L.) aleurone layer at fifteen stages of grain development. J. Proteom..

[63] Mahalingam R. (2018). Temporal Analyses of Barley Malting Stages Using Shotgun Proteomics. Proteomics.

[64] Rosenkilde A.L., Dionisio G., Holm P.B., Brinch-Pedersen H. (2014). Production of barley endoprotease B2 in Pichia pastoris and its proteolytic activity against native and recombinant hordeins. Phytochemistry.

[65] Diaz-Mendoza M., Dominguez-Figueroa J.D., Velasco-Arroyo B., Cambra I., Gonzalez-Melendi P., Lopez-Gonzalvez A., Garcia A., Hensel G., Kumlehn J., Diaz I. (2016). HvPap-1 C1A Protease and HvCPI-2 Cystatin Contribute to Barley Grain Filling and Germination. Plant Physiol..

[66] Bethune M.T., Khosla C. (2012). Oral enzyme therapy for celiac sprue. Methods Enzymol..

[67] Scherf K.A., Wieser H., Koehler P. (2018). Novel approaches for enzymatic gluten degradation to create high-quality gluten-free products. Food Res. Int..

[68] Hartmann G., Koehler P., Wieser H. (2006). Rapid degradation of gliadin peptides toxic for coeliac disease patients by proteases from germinating cereals. J. Cereal Sci..

[69] Kiyosaki T., Asakura T., Matsumoto I., Tamura T., Terauchi K., Funaki J., Kuroda M., Misaka T., Abe K. (2009). Wheat cysteine proteases triticain alpha, beta and gamma exhibit mutually distinct responses to gibberellin in germinating seeds. J. Plant Physiol..

[70] Savvateeva L.V., Gorokhovets N.V., Makarov V.A., Serebryakova M.V., Solovyev A.G., Morozov S.Y., Reddy V.P., Zernii E.Y., Zamyatnin A.A., Aliev G. (2015). Glutenase and collagenase activities of wheat cysteine protease Triticain-α: Feasibility for enzymatic therapy assays. Int. J. Biochem. Cell Biol..

[71] Cravatt B.F., Wright A.T., Kozarich J.W. (2008). Activity-based protein profiling: From enzyme chemistry to proteomic chemistry. Annu. Rev. Biochem..

[72] Lu H., Chandrasekar B., Oeljeklaus J., Misas-Villamil J.C., Wang Z., Shindo T., Bogyo M., Kaiser M., van der Hoorn R.A. (2015). Subfamily-Specific Fluorescent Probes for Cysteine Proteases Display Dynamic Protease Activities during Seed Germination. Plant Physiol..

[73] Daneri-Castro S.N., Chandrasekar B., Grosse-Holz F.M., van der Hoorn R.A., Roberts T.H. (2016). Activity-based protein profiling of hydrolytic enzymes induced by gibberellic acid in isolated aleurone layers of malting barley. FEBS Lett..

[74] Kok Y.J., Ye L., Muller J., Ow D.S., Bi X. (2019). Brewing with malted barley or raw barley: What makes the difference in the processes?. Appl. Microbiol. Biotechnol..

[75] Sánchez-León S., Gil-Humanes J., Ozuna C.V., Giménez M.J., Sousa C., Voytas D.F., Barro F. (2018). Low-gluten, nontransgenic wheat engineered with CRISPR/Cas9. Plant Biotechnol. J..

